# The Growing Unmet Need for Older Adult Nutrition Programs: Evidence from National Datasets

**DOI:** 10.1093/geroni/igaf122.391

**Published:** 2025-12-31

**Authors:** Bernadette Wright, Victoria Owens

**Affiliations:** Meals on Wheels America, Falls Church, Virginia, United States; Meals on Wheels America, Arlington, Virginia, United States

## Abstract

Home-delivered meal and community dining programs, such as Meals on Wheels, provide older adults with nutritious meals and the opportunity to address social and safety needs that affect health. With federal funding at risk and just 1% of philanthropic giving going to aging issues, nutrition programs need data to demonstrate the need. We examined trends over the past 25 years (based on availability) in service reach, funding and indicators of need. Data were from the National Health and Nutrition Examination Survey, Administration on Community Living, the Current Population Survey Food Security Supplement and the Health and Retirement Study. We found a stark disconnect between declining numbers of older adults served and growing indicators of need. 4.6 million Americans aged 60 and older reported receiving community or government home-delivered meals and/or community dining in the past 12 months (August 2021- August 2023), regardless of funding source. This was fewer people than in 6 of 10 prior periods since 1999-2000. 2.2 million older adults received Older Americans Act (OAA) Title III meal services in 2021, the fewest of any year since 1999. The purchasing power of OAA meal funding declined from $626 million in 1999 inflation-adjusted to $1.175 billion to $1.059 billion in FY2024. Meanwhile, the number of adults age 60 and older with food insecurity quadrupled between 1999 and 2023 (1.8 million to 7.4 million). 56% of older adults experienced loneliness in 2022, with little change since 2006. These findings suggest funding has not kept pace with the growing need.

